# Nicotinamide mononucleotide alleviates the streptozotocin-mediated ferroptosis of islet β-cells via the Nrf2/GPX4 pathway

**DOI:** 10.1590/1414-431X2026e15295

**Published:** 2026-08-17

**Authors:** Decheng Lu, Jianli Huang, Jinyue Meng, Jiayu Luo, Xuemei Huang

**Affiliations:** 1 Department of Endocrinology, The First Affiliated Hospital of Guangxi Medical University, Nanning, Guangxi, China; 2 Department of Endocrinology, The First People's Hospital of Nanning, Nanning, Guangxi, China; 3 Department of Endocrinology, The Fifth Affiliated Hospital of Guangxi Medical University, Nanning, Guangxi, China

**Keywords:** Nicotinamide mononucleotide, Islet β-cells, Ferroptosis, Nrf2/GPX4 pathway

## Abstract

The effects of nicotinamide mononucleotide (NMN) on streptozotocin (STZ)-induced islet β-cell dysfunction and the underlying mechanisms have not been fully elucidated. This study investigated the ameliorative effect and mechanism of NMN on islet β-cell dysfunction. NMN significantly increased the INS-1 cell viability following treatment with either STZ or erastin and effectively mitigated STZ-induced cell injury. Upon exposure to STZ, INS-1 cells exhibited significantly elevated intracellular Fe^2+^ levels, significantly increased reactive oxygen species (ROS) levels, and significantly decreased in the activities of superoxide dismutase (SOD) and glutathione (GSH). The levels of lipid peroxidation (LPO) and malondialdehyde (MDA) were substantially increased, and the mitochondrial membrane potential significantly decreased. Transmission electron microscopy further revealed mitochondrial shrinkage, rupture of the mitochondrial membrane, and disintegration of mitochondrial cristae. NMN markedly reduced intracellular iron accumulation in INS-1 cells treated with STZ. NMN subsequently attenuated intracellular ROS, LPO, and MDA levels while restoring GSH and SOD activities in STZ-exposed cells. Moreover, NMN restored the mitochondrial membrane potential and reversed the STZ-induced pathological changes in mitochondrial morphology. Compared with STZ treatment, ferrostatin-1 treatment significantly upregulated Nrf2 and GPX4 expression. In contrast, compared with STZ treatment, erastin treatment suppressed Nrf2 and GPX4 expression. Notably, compared with STZ or erastin treatment, cotreatment with NMN resulted in a further reduction in Keap1 expression and a significant upregulation of Nrf2 and GPX4. NMN may alleviate STZ-induced INS-1 cell ferroptosis by activating the Nrf2/GPX4 pathway, providing a theoretical basis for the treatment of diabetes mellitus.

## Introduction

Diabetes mellitus (DM) is a serious health problem worldwide, and its incidence is increasing annually. DM tends to increase the risk of related complications caused by macrovascular and microvascular damage, and it has negative effects on several organs, such as the heart, brain, kidney, and eyes. This not only exacerbates the financial burden of patients with DM but also places a heavy burden on public health systems worldwide (1-3). DM is characterized mainly by insulin resistance and islet β-cell dysfunction, and long-term glucotoxicity and lipotoxicity may lead to a progressive decline in islet β-cell function (4,5). Therefore, the regulation of islet β-cell function plays a critical role in the pathogenesis and progression of DM.

Ferroptosis is a novel type of programmed cell death that is characterized by intracellular iron overload and the accumulation of intracellular lipid peroxides. In addition, ferroptosis has unique morphological characteristics, mainly including a reduction in or disappearance of the mitochondrial crest, an increase in membrane density, and destruction of membrane integrity (6,7). Studies have revealed that ferroptosis plays a crucial role in the pathogenesis of DM and DM-related complications. Chen et al. (8) reported that ferroptosis in rat brain microvascular endothelial cells occurs after injury induced by oxygen and glucose deprivation combined with hyperglycemia. In addition, diabetes can cause severe myocardial ischemia perfusion injury by promoting ferroptosis in myocardial cells (9). Wang et al. (10) further demonstrated that ferroptosis occurs in the kidneys of diabetic mice and in proximal tubular epithelial cells cultured under high-glucose conditions. The role of the nuclear factor erythroid 2-related factor 2 (Nrf2)/glutathione peroxidase 4 (GPX4) pathway in ferroptosis has attracted considerable attention in recent years. Nrf2 serves as a critical regulator of cellular antioxidant responses, lipid peroxidation (LPO), and ferroptosis. It exerts its protective function by translocating into the nucleus to mitigate organ dysfunction. GPX4, a downstream target of Nrf2, is located in both the mitochondria and the cytoplasm, where it functions to eliminate lipid peroxides and thereby suppress ferroptosis (11,12). In the context of diabetic wounds, a bioactive compound derived from *Artemisia annua*, known as atemistine, has been shown to accelerate wound healing by inhibiting ferroptosis through activation of the Nrf2/GPX4 pathway (13). Ma et al. (14) demonstrated that proanthocyanidins ameliorate oxidative stress and attenuate ferroptosis via activation of the SIRT6/Nrf2/GPX4 signaling pathway, leading to improved trabecular bone architecture and increased expression of key osteogenic proteins in type 2 diabetic osteoporosis. Furthermore, hydrogen sulfide has been shown to alleviate cardiac damage in diabetic cardiomyopathy by regulating the Nrf2/GPX4/GSH pathway. This protective effect was mediated through the promotion of the Syvn1-Keap1 interaction, which reduced both ferroptosis and mitochondrial apoptosis in diabetic myocardial cells (15).

Nicotinamide mononucleotide (NMN) is an important precursor of nicotinamide adenine dinucleotide (NAD+) biosynthesis in mammals. NAD+ is an important oxidoreductase enzyme cofactor in eukaryotes that plays a key role in biological processes such as metabolism and DNA repair (16,17). NMN has been shown to protect against cerebrovascular and cardiovascular diseases by mediating oxidative stress and ferroptosis under pathological conditions. NMN was found to alleviate H_2_O_2_-induced oxidative stress damage in brain microvascular endothelial cells (18). Yagi et al. (19) reported that the ameliorating effects of NMN supplementation reduced the number of damaged lysosomes, thereby reducing heart failure in p32c KO mice. This effect was due to myocardial mitochondrial dysfunction-induced ferroptosis, which involved the accumulation of iron in lysosomes and lipid peroxidation. In addition, NMN restored lens architecture, reduced fibrosis, normalized the expression of the ferroptosis markers Fe2+ and malondialdehyde (MDA), and restored the expression of glutathione (GSH) and glutathione peroxidase 4 (GPX4) through the activation of the Nrf2/FPN1 pathway (20). However, the precise mechanism through which NMN regulates streptozotocin (STZ)-induced islet β-cell ferroptosis through the Nrf2/GPX4 pathway requires further investigation. Therefore, the primary objective of this study was to investigate whether NMN can activate the Nrf2/GPX4 pathway to alleviate STZ-induced INS-1 cell ferroptosis.

## Material and Methods

### Cell culture

INS-1 cells were obtained from the China Center for Type Culture Collection (cat. No. BNCC337862; China) and were cultured in RPMI-1640 medium (Gibco, Thermo Fisher Scientific, Inc., USA) supplemented with 10% fetal bovine serum (Gibco, Thermo Fisher Scientific, Inc.) and 1% penicillin-streptomycin solution (Solarbio, China). Mycoplasma testing was routinely carried out to ensure that the cell lines were mycoplasma free. The cells were incubated at 37°C in a 5% CO_2_ humidified atmosphere.

### Cell viability assay

Cell viability was measured by the Cell Counting Kit-8 (CCK-8) assay (Dojindo, Japan). Briefly, cells were plated at 5×10^3^ cells per well onto 96-well plates and incubated at 37°C and 5% CO_2_ for 24 h to achieve a confluence of 70-80%. The cells were treated with different concentrations of STZ (Solarbio) for 6 or 24 h. Then, the cells were washed with PBS and treated with erastin (MedChemExpress, China), ferrostatin-1 (Fer-1, MedChemExpress), or NMN (Sigma-Aldrich, USA) for 24 h. The cells were subsequently treated with 10 μL of CCK-8 reagent and incubated for 1 h at 37°C. The absorbance was measured at 450 nm using a microplate reader (Fluoroskan Ascent™; Thermo Fisher Scientific, Inc.), after which the cell viability was calculated based on the absorbance.

### Analysis of apoptosis

INS-1 cells were cultured in 6-well plates (3×10^6^ cells/well) for 24 h, treated with 0.25 mM STZ for 6 h, washed twice with PBS, and then treated with 0, 50, 100, or 200 µM NMN for 24 h. The cells were collected by trypsinization, washed twice with cold PBS, and then resuspended in 1× binding buffer. Afterward, 100 µL of the cell suspension was transferred to a 5-mL culture tube, followed by incubation with 5 µL of Annexin V-APC and 5 µL of 7-AAD (BD Biosciences, USA) in the dark for 15 min at room temperature. After 400 μL of binding buffer was added to each tube, apoptosis was analyzed with a FACS Calibur flow cytometer (BD Biosciences).

### Measurement of the intracellular Fe^2+^ concentration

FerroOrange (Dojindo) was used to measure the intracellular Fe^2+^ concentration according to the manufacturer's protocol. The cells were seeded onto 12-well plates (1×10^6^ cells/well), incubated at 37°C in 5% CO_2_ for 24 h, treated with 0.25 mM STZ for 6 h, washed twice with PBS, and then treated with 100 µM NMN for 24 h. The cells were subsequently washed with Hank's balanced salt solution (HBSS; Gibco, Thermo Fisher Scientific, Inc.) to remove the residual reagents. Afterward, the cells were treated with 1 µM FerroOrange in HBSS for 30 min at 37°C. The cells were observed and imaged with a fluorescence microscope (Nikon, Japan). The relative fluorescence intensities were calculated with ImageJ software version 1.8.0 (National Institutes of Health, USA).

### Measurement of ROS levels

To measure reactive oxygen species (ROS) levels, treated INS-1 cells were washed with serum-free culture medium and incubated with a 10 µM dichlorofluorescein diacetate (DCFH-DA) reaction mixture (Beyotime, China) for 30 min at 37°C. Afterward, the cells were washed twice with serum-free culture medium, and the fluorescence was measured in a microplate reader (Thermo Fisher Scientific) at an excitation wavelength of 488 nm and an emission wavelength of 525 nm.

### Measurement of oxidative stress-related enzyme activity and lipid peroxide content

Commercial kits were used to measure the concentrations of superoxide dismutase (SOD) (Nanjing Jiancheng, China), GSH (Nanjing Jiancheng), LPO (Solarbio), and MDA (Solarbio) in cells according to the manufacturers' instructions. The activity of these antioxidant enzymes was calculated on the basis of the absorbance.

### Measurement of mitochondrial membrane potential

The mitochondrial membrane potential was assayed via JC- according to the manufacturer's instructions (Beyotime). Briefly, cells were seeded onto 6-well plates at 3×10^6^ cells per well. After 24 h of growth, the cells were treated with 0.25 mM STZ for 6 h, washed twice with PBS, and treated with 100 µM NMN for 24 h. After staining with JC-1, the cells were analyzed via flow cytometry (BD Biosciences). The excitation wavelength of JC-1 was 488 nm, and the approximate emission wavelengths of the monomer and J-aggregate forms were 529 and 590 nm, respectively.

### Electron microscopy

INS-1 cells were treated with 0.25 mM STZ for 6 h, washed twice with PBS, and then treated with 100 µM NMN for 24 h. The cells were collected, washed twice with PBS, and fixed with phosphate buffer (pH 7.4) containing 2.5% glutaraldehyde overnight at 4°C. The cells were embedded in agarose, postfixed with 1% OsO_4_ at room temperature for 2 h, and dehydrated through a graded series of acetone. The resin blocks were cut into 60-80-nm sections on an ultramicrotome, fixed onto 150 mesh cuprum grids, and stained with 2% uranyl acetate. Images were taken under a transmission electron microscope (HT 7800; Hitachi Ltd., Japan) at a magnification of 3000× or 10000×.

### Total RNA extraction and RT-qPCR

INS-1 cells were treated with 0.25 mM STZ for 6 h, washed twice with PBS, and then treated with 100 µM erastin, 20 µM Fer-1, or 100 µM NMN for 24 h. Total RNA was extracted with TRIzol Reagent (Invitrogen, USA). The RNA was quantified spectrophotometrically at 260/280 nm. The RNA was reverse transcribed using PrimeScript^TM^ RT Master Mix (TaKaRa, China) according to the manufacturer's protocols, and mRNA expression was quantified via real-time PCR with a TB Green PrimeScript^TM^ RT-PCR Kit (TaKaRa) and an Applied Biosystems 7500 Real-Time PCR System (USA). The comparative Ct (2^-ΔΔCq^) value method was used to standardize the expression levels of all the target genes to that of the reference gene GAPDH (21). The primer sequences are listed in Table 1.

**Table 1:** Primer sequences of mRNA used in the study.

Gene	Forward primer (5'-3')	Reverse primer (5'-3')
*Keap1*	GGTCGCCCTGTGCCTCTAT	GACTAGGTGCCACTCGTCTCG
*Nrf2*	AAGACAAACATTCAAGCCGATT	TGAATTGCTCCTTGGACATCA
*GPX4*	CGAGTTCCTGGGCTTGTGTG	CACGCAACCCCTGTACTTATCC
*GAPDH*	GACATGCCGCCTGGAGAAAC	AGCCCAGGATGCCCTTTAGT

### Western blot analysis

INS-1 cells were treated with 0.25 mM STZ for 6 h, washed twice with PBS, and then treated with 100 µM erastin, 20 µM Fer-1, or 100 µM NMN for 24 h. The cells were gently washed with PBS three times. Total protein was subsequently extracted from the cells by incubating the cells in RIPA lysis buffer (Beyotime) containing the protease inhibitor phenylmethanesulfonyl fluoride (PMSF) on ice for 30 min. The cell lysate was transferred to a 1.5-mL centrifuge tube and centrifuged at 24,470 *g* for 15 min at 4°C, after which the supernatant was collected. The protein concentration was subsequently measured with a BCA protein assay kit (Beyotime). Protein denaturation was performed according to the protein concentration. A total of 30 µg of protein was separated via 10% SDS-PAGE and subsequently transferred to PVDF membranes. The membranes were blocked in TBST containing 5% skim milk for 1 h and incubated with specific primary antibodies at 4°C overnight [anti-Keap1 (1:1000; cat. No. 10503-2-AP; Proteintech, China), anti-Nrf2 (1:2000; cat. No. 16396-1-AP; Proteintech), anti-GPX4 (1:1000; cat. No. A11243; ABclonal, USA), and anti-GAPDH (1:10000; cat. No. 60004-1; Proteintech)]. After being washed with TBST 3 times, the membranes were incubated with horseradish peroxidase-conjugated secondary antibodies (1:5000; cat. No. SA00001-2; Proteintech) for 2 h at room temperature. The protein bands were visualized via a quantitative western blot imaging system (TOUCH IMAGER, eBlot, China) and analyzed via ImageJ software version 1.8.0. The intensity values of the protein bands were normalized to that of GAPDH. The experiment was repeated three times.

### Statistical analysis

Data are reported as means±SD and were analyzed using SPSS 26.0 software (SPSS Inc., IBM, USA). One-way ANOVA combined with the Bonferroni *post hoc* correction was used to analyze differences among sets of data. All the figures were created in GraphPad Prism 9.0 (GraphPad Software Inc., USA). Differences between groups were considered statistically significant at P<0.05.

## Results

### NMN alleviated STZ-induced INS-1 cell injury

**Figure 1:**
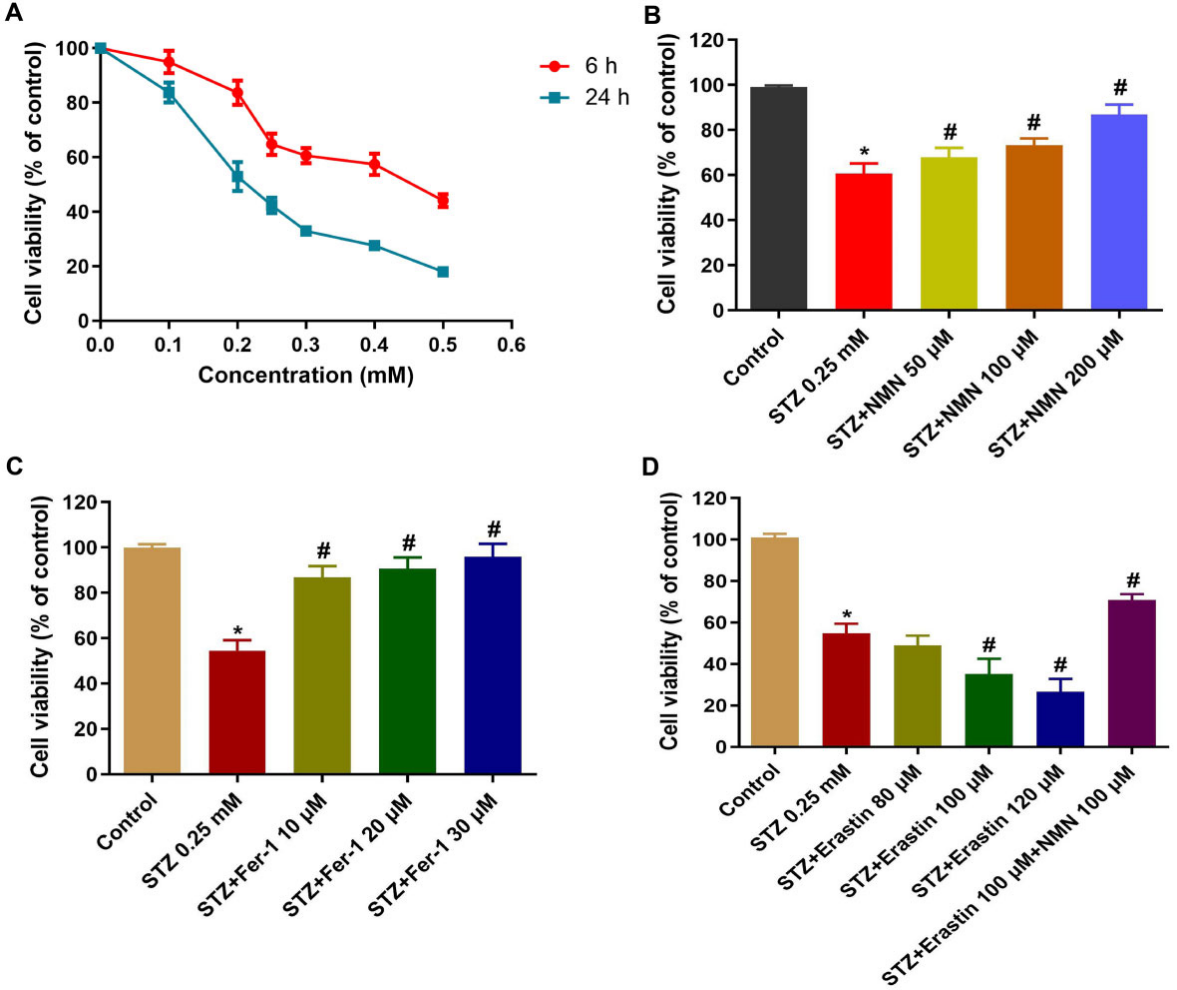
Nicotinamide mononucleotide (NMN) alleviated streptozotocin (STZ)-induced changes in INS-1 cell viability according to the CCK-8 assay. **A**, INS-1 cells were treated with various concentrations of STZ for 6 or 24 h. **B**, INS-1 cells were treated with 0.25 mM STZ for 6 h, and then treated with 0, 50, 100, or 200 µM NMN for 24 h. **C**, INS-1 cells were treated with 0.25 mM STZ for 6 h, and then treated with 10, 20, or 30 µM Fer-1 for 24 h. **D**, INS-1 cells were treated with 0.25 mM STZ for 6 h, and then treated with erastin (80, 100, or 120 µM) or 100 µM NMN for 24 h. Data are reported as means±SD (n=6). *P<0.05 compared with the control group; ^#^P<0.05 compared with the 0.25 mM STZ group (ANOVA).

INS-1 cells were incubated with various concentrations of STZ (0, 0.1, 0.2, 0.3, 0.4, and 0.5 mM) for 6 or 24 h (Figure 1A). CCK-8 assay results revealed that cell viability decreased significantly in a time- and concentration-dependent manner after STZ treatment. The viability of the INS-1 cells significantly decreased to 64.82±3.89% and 42.41±2.88% in the 0.25 mM STZ group at 6 and 24 h, respectively, and to 44.09±2.33% and 18.08±0.88% in the 0.5 mM STZ group at 6 and 24 h, respectively. We then selected INS-1 cells treated with 0.25 mM STZ for 6 h as the model for subsequent experiments in which the cells were treated with NMN. After the cells were treated with 0.25 mM STZ for 6 h, the survival rate of the cells decreased compared with that of the cells in the control group, and treatment with 50, 100, and 200 µM NMN for 24 h significantly increased the cell viability rate compared with that of the cells in the STZ group (Figure 1B).

After 6 h of pretreatment with 0.25 mM STZ, INS-1 cells were separately treated with the ferroptosis inhibitor Fer-1 (10, 20, or 30 µM) or the ferroptosis inducer erastin (80, 100, or 120 µM) for an additional 24 h. As illustrated in Figure 1C, Fer-1 treatment dose-dependently reversed STZ-induced cytotoxicity: compared with the STZ group, all three concentrations (10, 20, and 30 µM) significantly improved cell viability. In contrast, erastin exacerbated STZ-induced damage in a concentration-dependent manner; cell viability was further diminished in the groups treated with 100 and 120 µM erastin compared with the STZ group (Figure 1D). Notably, coadministration of NMN effectively attenuated the synergistic cytotoxic effect induced by the combination of STZ and erastin (Figure 1D).

**Figure 2:**
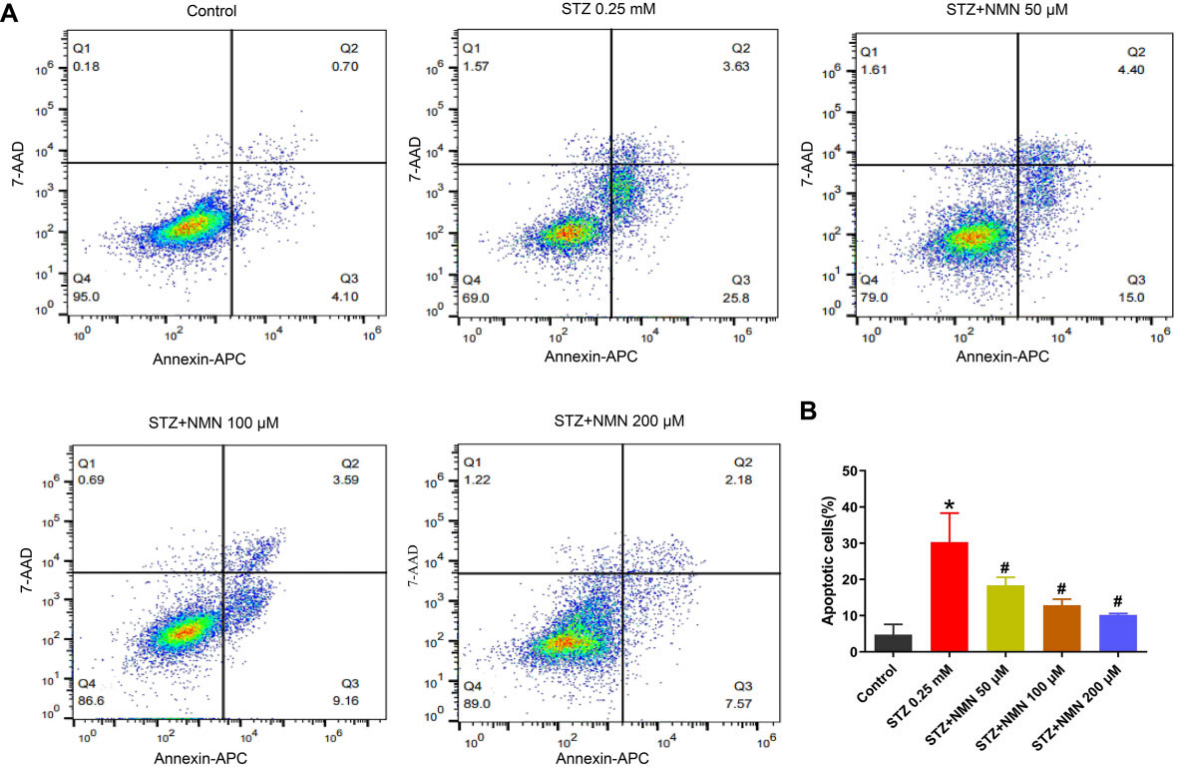
Nicotinamide mononucleotide (NMN) alleviated streptozotocin (STZ)-induced INS-1 cell apoptosis, as shown by flow cytometry. **A**, Flow cytometry assay was used to detect cell apoptosis (Q2: early apoptotic cells, Q3: late apoptotic cells). **B**, Quantification of INS-1 cell apoptosis. Data are reported means±SD (n=3). *P<0.05 compared with the control group; ^#^P<0.05 compared with the 0.25 mM STZ group (ANOVA).

We further verified the protective effect of NMN on STZ-induced INS-1 cell injury by flow cytometry. Among the INS-1 cells, 25.60±3.69% were apoptotic after exposure to 0.25 mM STZ for 6 h. Among the 50, 100, and 200 µM NMN-treated cells, 18.02±0.85%, 15.55±1.61%, and 10.72±0.49%, respectively, were apoptotic. The results indicated that the apoptosis rate of INS-1 cells significantly increased after STZ treatment but decreased after NMN treatment (P<0.05) (Figure 2).

### NMN alleviated STZ-induced ferroptosis in INS-1 cells

**Figure 3:**
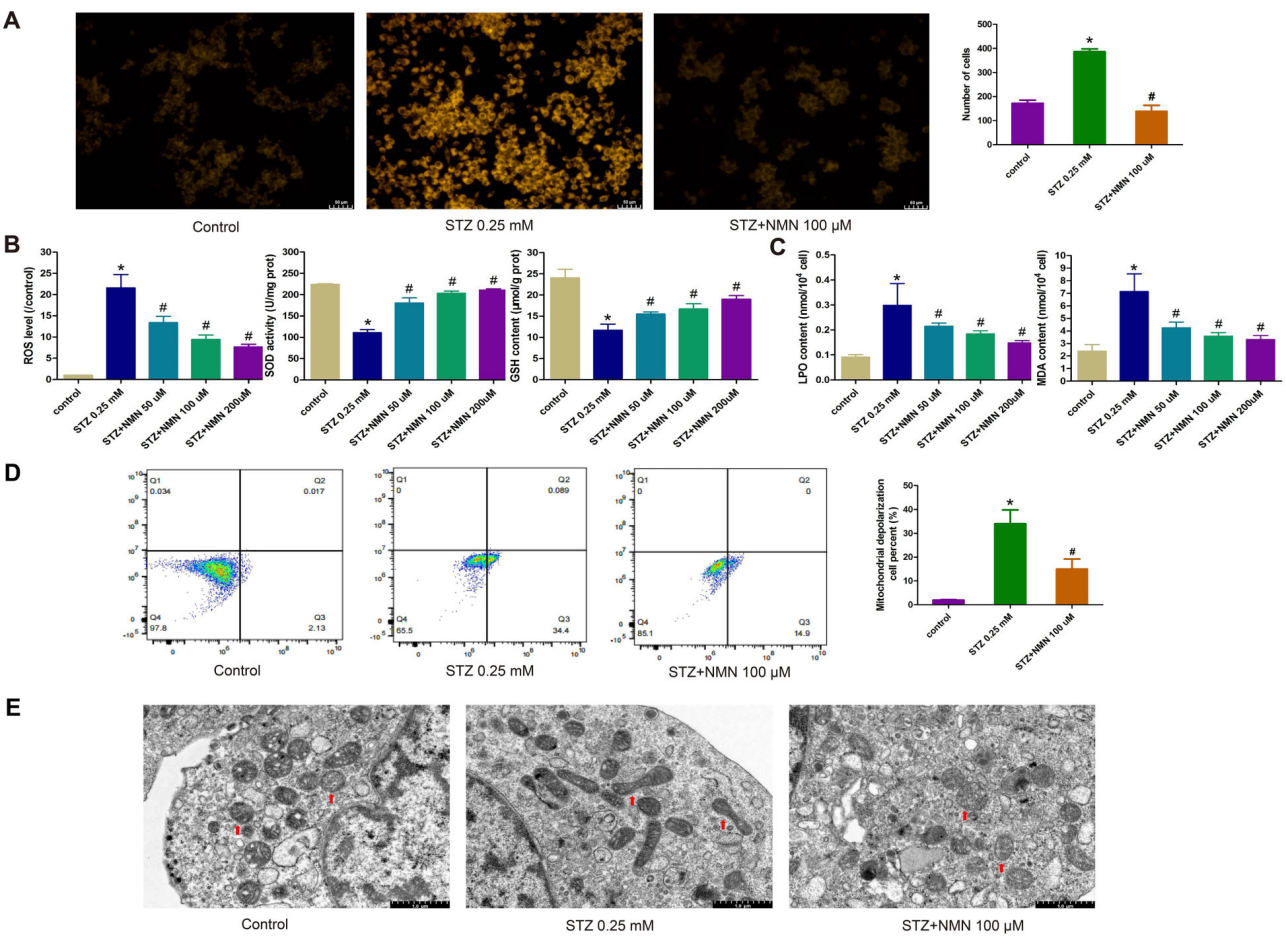
Nicotinamide mononucleotide (NMN) alleviated streptozotocin (STZ)-induced ferroptosis in INS-1 cells. **A**, Intracellular Fe^2+^ expression in INS-1 cells was detected by FerroOrange. Scale bars=50 μm. **B**, The levels of reactive oxygen species (ROS), superoxide dismutase (SOD), and glutathione (GSH) in the different groups. **C**, The levels of lipid peroxidation (LPO) and malondialdehyde (MDA) in the different groups. **D**, The proportion of cells mitochondrial depolarization was assayed via JC-1. **E**, Mitochondria (indicated by red arrows) were observed via transmission electron microscopy images showing representative mitochondrial structures in INS-1 cells. Scale bars=1.0 μm. Data are reported as means±SD (n=3). *P<0.05 compared with the control group; ^#^P<0.05 compared with the 0.25 mM STZ group (ANOVA).

INS-1 cells were treated with 0.25 mM STZ for 6 h, after which the accumulation of Fe^2+^ (yellow) was significantly altered, as determined via fluorescence microscopy. The Fe^2+^ concentration significantly increased in the STZ group. Moreover, 100 µM NMN significantly reduced the intracellular iron concentration in the STZ-treated INS-1 cells (Figure 3A). ROS levels increased significantly after 0.25 mM STZ treatment but decreased after NMN treatment. The activity of the important antioxidant SOD decreased by 50% after 0.25 mM STZ treatment, but this change was reversed by NMN treatment. The level of GSH was 0.49-fold lower in the STZ group than in the normal group but increased after treatment with NMN. NMN decreased the intracellular ROS levels but increased the SOD and GSH activities in the STZ-treated group (Figure 3B). The level of the lipid peroxide product LPO increased 3.29-fold after 0.25 mM STZ treatment but decreased after NMN treatment. In addition, the trend for the lipid peroxide product MDA was similar (Figure 3C).

Fluorescence staining of mitochondria with JC-1 dye revealed a significant increase in the proportion of cells exhibiting mitochondrial depolarization following 0.25 mM STZ treatment. NMN (100 µM) decreased the proportion of cells exhibiting mitochondrial depolarization (Figure 3D). In addition, compared with those in the control group, the mitochondria in the STZ-treated INS-1 cells were shrunken, and mitochondrial membrane rupture and mitochondrial crista destruction were observed via transmission electron microscopy. NMN led to the restoration of changes in mitochondrial morphology (Figure 3E).

### NMN alleviated STZ-induced INS-1 cell ferroptosis by activating the Nrf2/GPX4 pathway

**Figure 4:**
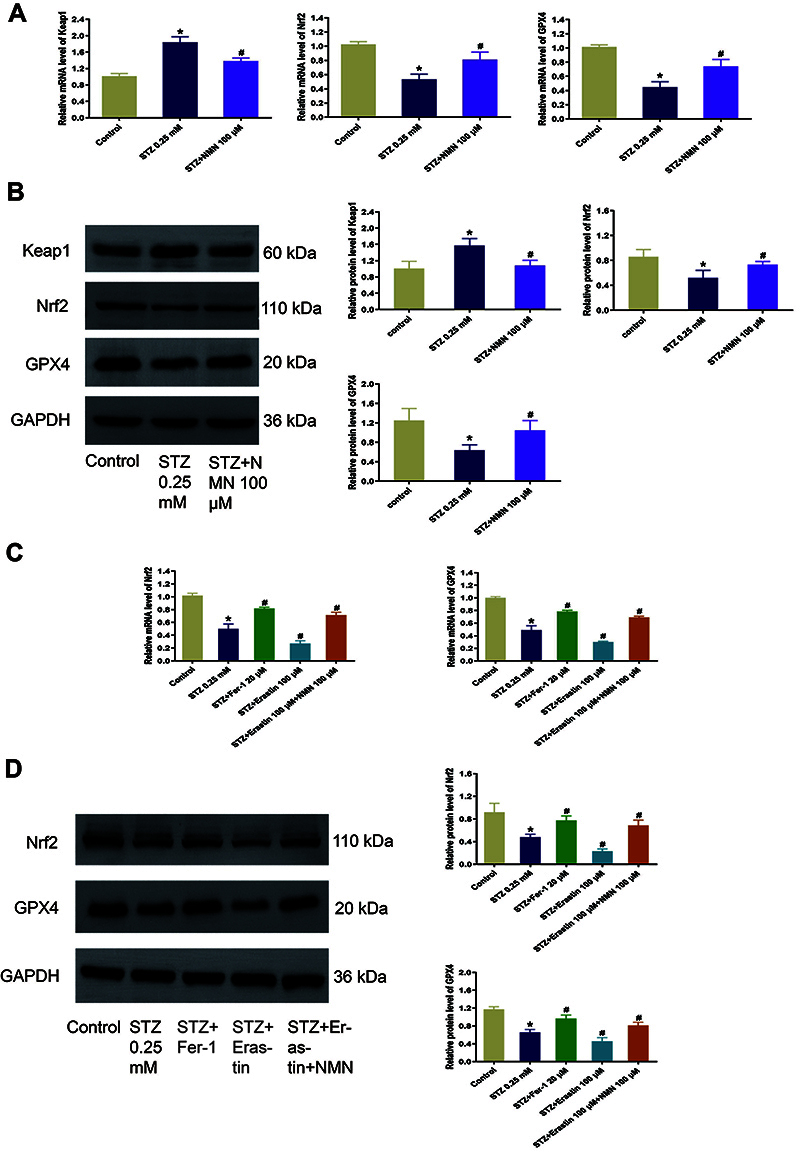
Nicotinamide mononucleotide (NMN) regulated streptozotocin (STZ)-induced ferroptosis in INS-1 cells by activating the Nrf2/GPX4 pathway. **A**, The mRNA expression levels of Keap1, Nrf2, and GPX4 were assessed by RT-qPCR, following treatment with STZ or NMN. **B**, The protein expression levels of Keap1, Nrf2, and GPX4 were assessed by western blot, following treatment with STZ or NMN. **C**, The mRNA expression levels of Nrf2 and GPX4 assessed by RT-qPCR, following treatment with STZ, Fer-1, erastin, or NMN. **D**, The protein expression levels of Nrf2 and GPX4 were assessed by western blot, following treatment with STZ, Fer-1, erastin, or NMN. Data are reported as means±SD (n=3). *P<0.05 compared with the control group; ^#^P<0.05 compared with the 0.25 mM STZ group (ANOVA).

The expression levels of the major genes of the Nrf2/GPX4 pathway related to ferroptosis, including Keap1, Nrf2, and GPX4, were analyzed (Figure 4A and B). Keap1 expression was greater in the 0.25 mM STZ group than in the control group, and there was a further reduction in Keap1 expression after treatment with NMN. The INS-1 cell data revealed that the expression of Nrf2 and GPX4 decreased after treatment with 0.25 mM STZ but increased after treatment with NMN.

To further substantiate the role of NMN in modulating INS-1 cell ferroptosis via the Nrf2/GPX4 pathway, we used pharmacological ferroptosis inducers and inhibitors. Following a 6-h preincubation with 0.25 mM STZ, INS-1 cells were treated for 24 h with either the ferroptosis inhibitor Fer-1 (20 µM) or the ferroptosis inducer erastin (100 µM). As expected, compared with the STZ treatment, Fer-1 treatment significantly upregulated Nrf2 and GPX4 expression. Conversely, compared with STZ treatment, erastin treatment suppressed Nrf2 and GPX4 expression. Importantly, compared with STZ + erastin treatment, treatment with NMN (100 µM, 24 h) led to a more pronounced upregulation of Nrf2 and GPX4 expression (Figure 4C and D). These results indicate that NMN alleviated STZ-induced ferroptosis in INS-1 cells through activating the Nrf2/GPX4 pathway.

## Discussion

To explore the mechanism underlying the effects of NMN on pancreatic islet β-cells, we established a model of STZ-induced islet β-cell injury. The results revealed that the damage to INS-1 cells gradually increased with increasing STZ concentration and time. However, NMN alleviated the STZ-induced damage to INS-1 cells, as determined by the CCK-8 and flow cytometry assays. An *in vitro* study revealed that NMN increased cell viability by reducing cell damage, reducing apoptosis, increasing cell migration, and restoring tight junctions in high glucose-treated human corneal epithelial cells (22). Tuncay et al. (23) reported that NMN improved the survival rate of cardiomyocytes and protected against ischemia or hypoxia-induced cardiomyocyte damage in a K_ATP_ channel-dependent manner. Furthermore, NMN promoted the growth of epidermal cells and vascular endothelial cells and accelerated the healing of diabetic foot ulcers (24). These findings indicate that NMN alleviates damage to β-cells.

Iron accumulation serves as both a catalyst and an amplifier of lipid peroxidation reactions during ferroptosis (25). In our study, compared with the control, treatment of INS-1 cells with STZ resulted in significant increases in the intracellular iron content and ROS levels, and a marked decrease in SOD activity. Excess iron catalyzes the Fenton reaction, thereby generating excessive amounts of ROS. As potent initiators of LPO, ROS trigger and propagate the lipid peroxidation chain reaction (26). MDA, a highly reactive aldehyde product of lipid peroxidation, serves as a widely accepted biomarker for membrane lipid peroxidation (27). SOD is a key antioxidant enzyme that scavenges superoxide radicals and mitigates oxidative stress (28). To assess lipid peroxidation reactions in INS-1 cells, we quantified both LPO activity and MDA levels. Consistent with ferroptotic progression, both the LPO and MDA levels were significantly elevated in the STZ-induced group compared with those in the control group. Critically, the interplay between iron overload and LPO establishes a self-amplifying positive feedback loop that accelerates ferroptosis (29). We further examined the STZ-induced alterations in membrane potential and mitochondrial morphology. The results revealed a significant increase in the proportion of cells exhibiting mitochondrial depolarization following STZ treatment. Transmission electron microscopy further demonstrated characteristic ultrastructural changes, including mitochondrial atrophy, rupture of the outer mitochondrial membrane, and a marked reduction or complete loss of cristae. Collectively, these morphological and functional impairments strongly indicate that STZ induces ferroptosis in INS-1 cells. Notably, NMN treatment markedly attenuated STZ-induced ROS accumulation and restored SOD activity. GSH, a critical intracellular antioxidant, was significantly depleted in the STZ group but was effectively replenished upon NMN administration. Similarly, the STZ-induced increases in LPO and MDA levels were significantly reversed by NMN treatment. Moreover, NMN ameliorated STZ-induced mitochondrial structural abnormalities and restored mitochondrial membrane potential in INS-1 cells. Tan et al. (30) identified divalent metal transporter 1 (DMT1) as a key regulator of mitochondrial membrane potential. NMN enhanced cellular antioxidant capacity during erastin-induced ferroptosis and conferred protection against ferroptosis by increasing GSH levels, a mechanism phenocopying the effects of DMT1 deficiency. Ma et al. (31) demonstrated that macrophages isolated from aged mice exhibited heightened susceptibility to ferroptosis, a phenotype that was effectively reversed upon NMN supplementation. Furthermore, cotreatment with the ferroptosis inducer erastin and STZ significantly reduced INS-1 cell viability; however, NMN reduced the decrease in INS-1 cell viability induced by this combination. Our study revealed that NMN alleviated ferroptosis in INS-1 cells.

**Figure 5:**
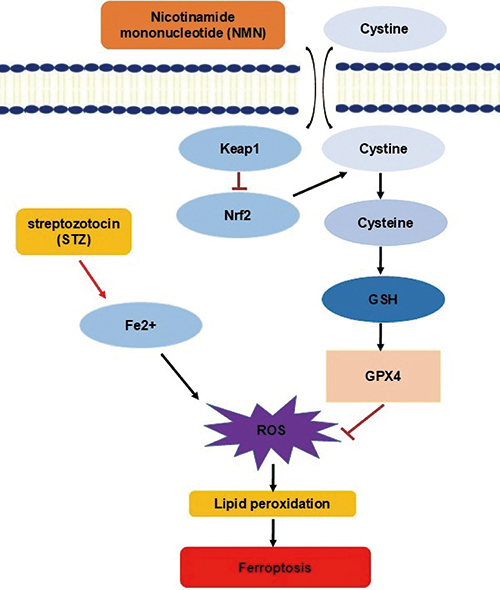
The role of nicotinamide mononucleotide (NMN) in regulating ferroptosis in INS-1 cells.

The Nrf2/GPX4 pathway has been identified as a critical signaling pathway involved in cellular antioxidant defense and ferroptosis. Under normal physiological conditions, Nrf2 is specifically bound to Keap1 in the cytoplasm. In response to oxidative stress, Nrf2 dissociates from Keap1 and promotes the entry of extracellular cystine into cells, where it is subsequently converted to GSH. GPX4 can use GSH to decrease lipid hydroperoxide levels, thereby inhibiting ferroptosis (15,32). In our study, STZ treatment significantly increased Keap1 expression in INS-1 cells. Concurrently, the levels of both Nrf2 and GPX4 were markedly decreased following STZ exposure, whereas NMN administration effectively restored their expression. Consistent with these findings, Liu et al. (33) demonstrated that NMN treatment reversed Keap1 accumulation and Nrf2 depletion in the hippocampus of D-galactose-induced aging rats. Moreover, NMN has been shown to increase GPX4-mediated ferroptosis defense by promoting the recruitment of GSH during UV irradiation-induced skin injury, thereby mitigating oxidative skin damage (34). Similarly, NMN alleviated silica-induced pulmonary injury in mice by activating the Nrf2-dependent transcriptional program to increase GSH synthesis and scavenge ROS (35). To further investigate the functional relevance of this pathway in ferroptosis regulation, we pretreated INS-1 cells with STZ, followed by treatment with either the ferroptosis inhibitor Fer-1 or the ferroptosis inducer erastin. Compared with STZ treatment, Fer-1 treatment significantly upregulated both Nrf2 and GPX4 expression. In contrast, compared with STZ treatment, erastin further suppressed Nrf2 and GPX4 expression. Notably, compared with treatment with erastin + STZ, treatment with NMN led to a greater restoration of Nrf2 and GPX4 expression. Collectively, these results indicate that NMN inhibited ferroptosis in INS-1 cells by activating the Nrf2/GPX4 pathway (Figure 5).

## Conclusions

In summary, the results of the present study indicate that NMN has the potential to ameliorate STZ-induced islet INS-1 ferroptosis through the activation of the Nrf2/GPX4 pathway. However, certain limitations remain. In this study, rat islet INS-1 cells were used to explore the underlying mechanism through which NMN affects islet β-cells. Validation data from mouse islet cell lines, such as NIT-1 and MIN6, are currently lacking. Further studies are needed to confirm the mechanistic effects of NMN on islet β-cells using diabetic animal models. Nevertheless, the findings of this study offer valuable insights into the potential therapeutic application of NMN in the management of DM.

## Data Availability

All data generated or analyzed during this study are included in this published article.
